# N-Acetylcysteine Prevents Skeletal Muscle Cisplatin-Induced Atrophy by Inducing Myogenic microRNAs and Maintaining the Redox Balance

**DOI:** 10.3390/antiox14111344

**Published:** 2025-11-08

**Authors:** Teminijesu Dorcas Aremu, Tonali Blanco Ayala, Karla F. Meza-Sosa, Daniela Ramírez Ortega, Dinora F. González Esquivel, Gustavo I. Vázquez Cervantes, Itamar Flores, Wendy Leslie González Alfonso, Verónica Custodio Ramírez, Alelí Salazar, Benjamin Pineda, Gonzalo Pérez de la Cruz, Saul Gómez Manzo, Gabriel Roldan Roldan, Paul Carrillo Mora, Verónica Pérez de la Cruz

**Affiliations:** 1 Neurochemistry and Behavior Laboratory, National Institute of Neurology and Neurosurgery “Manuel Velasco Suárez”, Mexico City 14269, Mexico; ojuadeteminijesu@gmail.com (T.D.A.); tblanco@innn.edu.mx (T.B.A.); karla.meza@innn.edu.mx (K.F.M.-S.); danielaramirez@innn.edu.mx (D.R.O.); dinora.gonzalez@innn.edu.mx (D.F.G.E.); 2 Doctorado en Ciencias Biológicas, Posgrado en Ciencias Biológicas, Coordinación de la División de Ciencias Biológicas, Centro Tlaxcala de Biología de la Conducta, Universidad Autónoma de Tlaxcala, Tlaxcala 90070, Mexico; 3 Laboratorio de Neurobiología Conductual, Departamento de Fisiología, Facultad de Medicina, Universidad Nacional Autónoma de México (UNAM), Mexico City 04510, Mexico; gabaergico@gmail.com; 4 Neuroimmunology Laboratory, National Institute of Neurology and Neurosurgery “Manuel Velasco Suárez”, Mexico City 14269, Mexico; gustavo.vazquez@innn.edu.mx (G.I.V.C.); itamar.flores@innn.edu.mx (I.F.); wendy.gonzalez@innn.edu.mx (W.L.G.A.); aleli.salazar@innn.edu.mx (A.S.); 5 Experimental Laboratory of Neurodegenerative Diseases, National Institute of Neurology and Neurosurgery “Manuel Velasco Suárez”, Mexico City 14269, Mexico; vcustodio@innn.edu.mx; 6 Department of Mathematics, Faculty of Sciences, Universidad Nacional Autónoma de México (UNAM), Mexico City 04510, Mexico; gonzalo.perez@ciencias.unam.mx; 7 Laboratorio de Bioquímica Genética, Instituto Nacional de Pediatría, Secretaría de Salud, Mexico City 04530, Mexico; saulmanzo@ciencias.unam.mx; 8 Clinical Neurosciences Division, National Institute of Rehabilitation “Luis Guillermo Ibarra Ibarra”, Mexico City 14389, Mexico; pcarrillo@inr.gob.mx

**Keywords:** myogenic microRNAs, myomiRs, redox environment, skeletal muscle

## Abstract

Cisplatin (CIS) is a widely used chemotherapeutic agent known for its efficacy; however, it induces several adverse effects, most notably cachexia, which is characterized by progressive loss of skeletal muscle mass, weakness, and reduced body weight. N-acetylcysteine (NAC) a compound with antioxidants properties, has been shown to mitigate CIS-induced neurotoxicity in experimental models. This study aimed to investigate the myoprotective effects of NAC during CIS treatment and explore the redox and molecular mechanisms involved in this response. For this, female Wistar rats were divided into four experimental groups: Control, NAC (300 mg/day/8 days), CIS (3 mg/kg i.p for 5 days), and NAC + CIS (NAC for 8 days, with CIS administered from day 4 onward). After treatment, muscle strength, redox status, mitochondrial biogenesis, expression of myogenic microRNAs and morphological changes were evaluated. CIS treatment caused muscle atrophy, decreased GSH/GSSG ratio, impaired cellular function, increased lipid peroxidation and altered antioxidant enzymes activity. These effects were mitigated by NAC coadministration. CIS also reduced the mtDNA/nDNA ratio; however, NAC treatment tended to increase TFAM and PGC-1α expression levels. Furthermore, CIS suppressed the expression of muscular miR-1-3p, miR-133a-3p and miR-206-3p, while NAC restored their levels when co-administered with CIS. These findings suggest that NAC may serve as a promising adjuvant therapeutic strategy to counteract CIS-induced myotoxicity through redox regulation and modulation of molecular pathways related to muscle integrity and regeneration.

## 1. Introduction

Cancer remains one of the leading causes of morbidity and mortality worldwide, with its incidence expected to rise significantly in the coming decades [1]. Despite considerable advances in early diagnosis and targeted therapies, chemotherapy continues to be a cornerstone in the management of both solid and hematologic malignancies. Among the most widely used chemotherapeutic agents is cisplatin (CIS), a platinum-based compound that exerts its cytotoxic effect primarily through the formation of DNA crosslinks, leading to genetic damage, cell cycle arrest, and apoptosis [2]. Due to its efficacy, CIS is a first-line treatment for a variety of cancers, including the most prevalent types of cancer in women, such as breast and cervical cancer [2,3]. However, its clinical application is frequently limited by a spectrum of adverse effects, such as nephrotoxicity, neurotoxicity, ototoxicity and, more recently acknowledged, chemotherapy-induced cachexia [4,5,6].

Cachexia is defined as a progressive and involuntary loss of skeletal mass, typically accompanied by fatigue, weakness, and significant body weight reduction [7,8]. It is estimated to affect up to 80% of patients with advanced-stage cancer and is associated with poor clinical outcomes. Cachexia not only deteriorates the patients’ functional status, but it also reduces treatment tolerance and significantly decreases overall survival [9,10].

Experimental models have shown that CIS administration in healthy rats reproduced many of the clinical features of cachexia, including weight loss, adipose tissue remodeling, muscle atrophy, reduction in myofiber diameter, and decreased muscle strength [11,12,13,14,15,16]. Studies in rodents have reported that administering CIS at doses of 1–3 mg/kg over 3–4 consecutive days can lose to up to 30% of total body weight [13,17,18]. The underlying mechanisms of CIS-induced muscle atrophy are multifactorial and some of the already reported include increased oxidative stress, activation of the ubiquitin-proteasome system, and impaired mitochondrial function in skeletal muscle [16,17,19].

In a previous study from our group, we demonstrated that CIS administration in female rats induced not only cognitive impairments (a phenomenon referred to as “chemobrain”) but also significantly reduce rats’ performance in the wire hang test, suggesting a functional decline in skeletal muscle unrelated to motivational or cognitive factors [20]. Notably, co-administration of N-acetylcysteine (NAC) attenuated these effects, highlighting its therapeutic potential in the central nervous system and in the musculoskeletal system.

NAC is a clinically approved and well-tolerated drug, commonly used as a mucolytic agent and as an antidote for acetaminophen overdose. Its use has expanded due to its ability to promote endogenous antioxidant defense, acting as a precursor to reduced glutathione (GSH), a key regulator of the cellular redox state. Moreover, NAC is a scavenger of reactive oxygen species (ROS) and a supplier of Krebs cycle intermediates, thereby supporting mitochondrial bioenergetics [21,22]. Its protective effects have been demonstrated in several studies, showing that NAC can reduce CIS-induced nephrotoxicity, ototoxicity, cardiotoxicity, and alopecia, effects primarily attributed to its role as a modulator of redox homeostasis [23,24,25,26].

Previous clinical studies suggest that NAC decreases post-exercise fatigue and soreness, improves muscular strength, and to reduces inflammation markers in healthy subjects [27,28,29,30]. However, whether NAC has a therapeutic role in the toxic muscular effects of CIS and its influence on the expression of muscular microRNAs (miRNAs) remains underexplored. miRNAs are a class of small non-coding RNAs that negatively regulate gene expression at the post-transcriptional level and are essential for various biological processes, including cell proliferation, differentiation, apoptosis and stress response [31,32,33]. Based on its critical role as regulators of muscle development, maintenance and regeneration, 8 miRNAs (miR-1, miR-133a/b, miR-206, miR-208a/b, miR-486 and miR-499) have been denominated as myogenic miRNAs (myomiRs) [34]; and alterations in their expression could be associated with muscle atrophy and mitochondrial dysfunction [35,36]. Therefore, in this study, we aimed to investigate whether NAC can prevent CIS-induced skeletal muscle atrophy and to elucidate some of the underlying redox and molecular mechanisms involved in this process.

## 2. Materials and Methods

### 2.1. Animals

Female Wistar rats (200–250 g) were used for all experiments. Animals were housed in standard acrylic cages (8 animals per cage) and maintained under controlled environmental conditions: temperature (25 ± 3 °C), relative humidity (50 ± 10%) and 12:12 h light-dark cycle. Rats had ad libitum access to a standard rodent chow diet and filtered drinking water throughout the study. All procedures were conducted in strict accordance with the Guide for the Care and Use of Laboratory Animals (National Research Council, 2011 [37]), and the experimental protocol approved by the Institutional Animal Care and Use Committee (Protocol 31/24, date of approval 17 May 2024). Efforts were made to minimize suffering during all procedures, particularly during tissue collection.

### 2.2. Experimental Design and Treatments

Animals were randomly assigned into four experimental groups: (1) Control group, received standard diet and intraperitoneal (i.p.) injections of physiological saline solution; (2) NAC group, received NAC (300 mg/kg/day, through NAC pellets) for 8 consecutive days; (3) CIS group, received cisplatin (3 mg/kg/day, i.p.) for 5 consecutive days; and (4) NAC + CIS group, received NAC for 8 consecutive days, with CIS administration starting on day 4 of the NAC treatment and continuing for 5 days. The selected treatment schedules were based on our prior work [20].

At the end of the treatment period, functional performance was evaluated through a standardized muscle strength test, and skeletal muscle tissue was harvested for further analysis. The collected tissue was used to assess muscle morphology, redox homeostasis, mitochondrial biogenesis markers and expression of myomiRs (miR-1-3p, miR133a-3p and miR-206-3p) and some of their target genes (*Cx43*, *Hdac4*, and *Srf*). Tissue samples were processed immediately or stored at −80 °C until further molecular and biochemical analyses were performed.

### 2.3. Assessment of Muscle Functional Performance

Assessment of muscle functionality was conducted 24 h after the last dose of CIS, during the light cycle (between 10:00 and 16:00 h), to minimize circadian variability. All animals were randomly assigned to their respective treatment groups to reduce allocation bias. Functional outcomes focused on forelimb muscle strength, were assessed using two complementary tests: the wire hang test and the measurement of digital grip strength.

#### 2.3.1. Forelimb Grip Endurance: Wire Hang Test

To determinate forelimb muscular endurance, a modified version of the wire hang test adapted from Ruan and Yao protocol was implemented [38]. Briefly, a stainless-steel bar (3 mm diameter, 50 cm in length) was horizontally suspended 50 cm above a padded surface to prevent injury from falls. Each rat was gently placed on the bar, allowing it to grasp it with its forelimbs and hang by its own strength. The latency to fall (in seconds) was recorded using a digital stopwatch. Each animal underwent three trials, separated by 1 min rest intervals, and the mean latency time was used as the outcome measurement.

#### 2.3.2. Quantitative Forelimb Strength

Forelimb strength was also quantitatively evaluated using a digital force gauge (also known as a dynamometer or grip strength meter). Each rat was gently held by the base of the tail and guided toward the device’s horizontal grip bar. Once the animal firmly grasped the bar with its forepaws, a consistent horizontal force was applied by gently pulling the tail backward until the grip was released. The maximum generated force (in Newtons) was automatically recorded by the device. Each animal underwent three consecutive trials, spaced by 1 min, and the mean of the three values was used for the statistical analysis [39].

### 2.4. Lipid Peroxidation

Lipid peroxidation (LP) was evaluated as a marker of oxidative stress in skeletal muscle. Triceps muscles were harvested from each experimental group and homogenized in 500 µL of Krebs buffer (pH 7.4) containing 19 mM NaCl, 5 mM KCl, 2 mM CaCl_2_, 1.2 mM MgSO_4_, 5 mM glucose, 13 mM NaH_2_PO_4_, and 3 mM Na_2_HPO_4_. LP levels were quantified by measuring thiobarbituric acid-reactive substances (TBARS), following a modified protocol based on the reaction with malondialdehyde (MDA), a byproduct of LP [20]. Briefly, 125 µL of muscle homogenate were mixed with 250 µL of TBA reagent, consisting of 0.375 g of thiobarbituric acid, 15 g of trichloroacetic acid (TCA), and 2.54 mL of hydrochloric acid (HCl), dissolved into 100 mL. To trigger the reaction the mixture was incubated in a boiling water bath for 15 min, then rapidly chilled on ice and spun at 9800× *g* for 15 min. The absorbance of the supernatant was measured at 532 nm using a Sinergy H1 microplate reader (Biotek-Agilent, Santa Clara, CA, USA). Results were expressed as µmoles of MDA per milligram of total protein.

#### Ex Vivo Prooxidant Susceptibility Assay

To assess susceptibility to oxidative stress, muscle homogenates from the Control and NAC groups were incubated ex vivo in 20 µM ferrous sulfate (FeSO_4_) in a separate set of assays. Brefly, a volume of 250 µL of homogenate were incubated in FeSO_4_ (20µM) at 37 °C for 2 h, and then, 250 µL of TBA reagent were added. Samples were then boiled, spun and processed identically to those of the in vivo TBARS assay. The degree of LP was represented as a percentage of the untreated control.

### 2.5. Determination of the GSH/GSSG Ratio

Muscle tissue (20–30 mg) was homogenized (1:10, *p*/*v*) in Buffer A (154 mM KCl, 5 mM DTPA, and 0.1 M potassium phosphate buffer; pH 6.8) and mixed 1:1 with Buffer B (20 mM ascorbic acid, 10 mM DTPA, 40 mM HCl, and 10% TCA). Samples were centrifuged at 14,000× *g* for 20 min, and supernatants were filtered (0.45 µm). For GSH determination, samples were treated with OPA according to Senft [40]. For GSSG detection, GSH was neutralized with NEM (7.5 mM), reduced to GSH using 100 mM sodium hydrosulfite, and measured as an isoindole when OPA was added. Products were measured by fluorescence at 365 nm excitation/430 nm emission in a Synergy H1 microplate reader (Biotek-Agilent, Santa Clara, CA, USA). The GSH/GSSG ratio was calculated for each sample.

### 2.6. Cellular Function Assay

Cellular metabolic activity was assessed using a 3-(4,5-dimethylthiazol-2-yl)-2,5-diphenyltetrazolium bromide (MTT) assay, which evaluated dehydrogenases-dependent reduction in MTT to formazan salts. Muscle tissue was homogenized (1:10) in Krebs-Ringer buffer (118.5 mM NaCl; 4.75 mM KCl; 1.77 mM CaCl_2_; 1.18 mM MgSO_4_; 5 mM glucose; 12.9 mM NaH_2_PO_4_ and 3 mM Na_2_HPO_4_; pH 7.4). 100 µL were incubated in 4 μL of MTT (5 mg/mL) on a shaking water bath at 37 °C for 15 min. Then, samples were placed on ice for 5 min and then centrifuged at 19,000× *g* for 3 min. The supernatants were discarded, and the pellets with the formazan salts were properly dissolved in 250 μL of acidic isopropanol. 300 µL of the colored formazan product were measured spectrophotometrically at 570 nm emission in a Synergy H1 microplate reader (Biotek-Agilent, Santa Clara, CA, USA).

### 2.7. Glutathione Reductase (GR) Activity

To determinate GR enzymatic activity, 30 mg of skeletal muscle tissue were homogenized in 200 µL of homogenization buffer (50 mM phosphate buffer, pH 7; 0.05% Triton X-100). Then, samples were centrifuged at 10,000× *g* for 30 min and supernatants were obtained. GR activity was assessed by mixing 16.5 μL of the corresponding supernatant with 300 μL of reaction cocktail (0.5 mM EDTA; 1 mM NADPH; 2.5 mM GSSG). The optic density was immediately measured at 340 nm, every minute for 3 min, in a Sinergy H1 microplate reader (Agilent, Santa Clara, CA, USA). Results were expressed as Units of GR per protein milligram.

### 2.8. Glutathione Peroxidase Activity (GPx)

GPx activity was evaluated in skeletal muscle supernatants obtained as previously described for GR. Supernatants (25 μL) were mixed in 200 μL of reaction cocktail (1 mM GSH; 0.2 mM NADPH; 1 U/mL glutathione reductase), and with 100 μL of 0.25 mM H_2_O_2_. The optical density was immediately measured at 340 nm, every 30 s for 5 min usimg a Sinergy H1 microplate reader (Agilent, Santa Clara, CA, USA). Results were expressed as Units of GPx per protein milligram.

### 2.9. Relative Mitochondrial DNA Content

20 mg of skeletal muscle tissue from rats of each experimental group were incubated in lysis buffer (100 mM Tris, 200 mM NaCl, 25 mM EDTA, 0.5% pH 8.5 SDS, and 0.2 mg/mL proteinase K) at 55 °C for 16 h. Next, samples were treated with 0.3 mg/mL RNAse A at 37 °C for 30 min, and then, DNA was purified by the isopropanol precipitation method. After that, the mitochondrial DNA (mtDNA)/nuclear DNA (nDNA) ratio was determined by qPCR using the next oligonucleotides: Mito-Fwd: GGTTCTTACTTCAGGGCCATCA and Mito-Rev: TGATTAGACCCGTTACCATCGA for measuring mtDNA; and β-actin-Fwd: CCCAGCCATGTACGTAGCCA and β-actin-Rev: CGTCTCCGGAGTCCATCAC to take β-actin’s copy number as the known nuclear DNA parameter. The 2X Brilliant III Ultra-Fast SYBR Green qPCR Master Mix with Low ROX (Agilent: 600892, Agilent Technologies, Santa Clara, CA, USA) and a 7500 Real Time PCR system thermocycler (Applied Biosystems, Waltham, MA, USA) were used following manufacturer instructions. Finally, the mtDNA/nDNA ratio was calculated with the 2^−ΔCt^ method, in which the difference between the Ct values of the mitochondrial gene and *β-actin* determined the relative abundance of mtDNA.

### 2.10. Western Blotting

To extract soluble proteins, muscle tissue was homogenized in RIPA buffer (Santa Cruz Biotechnology, Santa Cruz, CA, USA, Cat No sc-24948A) by sonication using an amplitude of 30%. Samples were then spun at 16,000× *g* for 20 min at 4 °C, and supernatants collected for Western blot. Protein concentration was determined using the Bradford microplate assay (Bio-Rad, Hercules, CA, USA) and a standard curve of the bovine serum albumin (BSA) protein (R2  >  0.98). 50 μg of protein were loaded in 10% SDS-acrylamide gels and transferred to nitrocellulose membranes (Bio-Rad Laboratories, Hercules, CA, USA). Ponceau staining was used as a protein loading control. Membranes were blocked with Tris-buffered saline solution containing 5% non-fat milk and 0.1% Tween-20 for 1 h. Membranes were then incubated with the respective primary antibodies overnight at 4 °C, followed by incubation with the corresponding secondary antibodies. The used antibodies were: rabbit polyclonal antibody for anti-PGC1 alpha Antibody (Abcam, Cambridge, UK, Cat No. ab-191838, dilution: 1/500) and HRP goat anti-rabbit IgG (LI-COR Biosciences, Lincoln, NE, USA, Cat No 92680011, dilution: 1/10,000). Protein bands were revealed using the NZY Advanced ECL (NZYtech, Lisboa, Portugal, Cat No. MBA40201). A UVP Chem Studio equipment (Analytic Jena, Jena Germany) was used for imaging. Images were analyzed using the 1.54 g version of the ImageJ software (US National Institutes of Health, Bethesda, MD, USA). Relative levels of protein were determined by normalizing the optical density of each band by the signal obtained for the loading control.

### 2.11. Small Non-Coding RNAs (sncRNAs) and Messenger RNAs (mRNAs) Reverse Transcription (RT) and Quantitative PCR (qPCR)

Muscle tissue was homogenized in 200 μL of Trizol reagent (ThermoFisher: 15596026, Waltham, MA, USA) and then, total RNA extraction was performed following the manufacturer’s directions. RNA concentration was determined using a NanoDrop 2000/2000c spectrophotometer (ThermoFisher: ND-2000C). A multiplex method was standardized to simultaneously perform the reverse transcription (RT) of 3 miRNAs (miR-1-3p, miR-133a-3p, and miR-206-3p) and the small nucleolar RNA (snoRNA), U6. For this, a specific stem-loop RT primer was designed for each miRNA of interest following a previously reported protocol [41]. Briefly, 100 nanograms (ng) of total RNA were mixed with 0.25 μL of the Revertaid reverse transcriptase (ThermoFisher: EP0441), 0.5 μL of 10 mM dNTPs mix solution (ThermoFisher: R0192), 1 μL of 1 µM of each stem-loop RT primer of interest (multiplex approach), 1 μL of 1 µM U6 reverse primer, 4 μL of 5x RT buffer, and nuclease-free water up to 20 μL. RT reactions were incubated at 16 °C for 30 min, 42 °C for 60 min, and at 70 °C for 10 min. U6 and miRNAs’ stem-loop RT primers are listed in Table 1. For longer transcripts (*Cx43*, *Hdac4*, *Srf*, and *β-actin*), 500 ng of total RNA were mixed with 0.25 μL of the Revertaid reverse transcriptase (ThermoFisher: EP0441), 1 μL of 10 mM dNTPs mix solution (ThermoFisher: R0192), 1 μL of 50 µM oligo(dT)15, 0.5 μL of 50 µM random hexamers (ThermoFisher: N8080127), 4 μL of 5× RT buffer and nuclease-free water up to 20 μL. RT reactions were incubated at 25 °C for 10 min, 42 °C for 60 min, and at 70 °C for 10 min. Complementary DNAs (cDNAs) for both small non-coding RNAs (miRNAs + U6) and regular genes were diluted at 1:10 and stored at −20 °C until use.

10 μL qPCR reactions were prepared by mixing 4.5 μL of the corresponding diluted cDNA, 5 μL of the 2X Brilliant III Ultra-Fast SYBR Green qPCR Master Mix with Low ROX (Agilent: 600892), and 0.5 μL of 10 µM oligo mix to amplify either each miRNA, each regular gene or U6. qPCRs were performed using an AriaMx real-time Thermalcycler (Agilent Technologies, Santa Clara, CA, USA) following the next program: 1 cycle at 95 °C for 10 min, 40 cycles at 95 °C for 15 s, and 1 cycle at 60 °C for 1 min. Then, melt curves were obtained like this: 95 °C for 15 s; 60 °C either for 30 s (for miRNAs and U6) or for 1 min (for regular genes), and finally, going from 60 °C to 95 °C with a 0.5 °C increase every 5 s. Individual Ct values were used to calculate relative expression levels of miRNAs (normalized to U6) and regular genes (normalized to β-actin) using the 2^−∆∆Ct^ method. All used qPCR primers are listed in Table 1.

### 2.12. Histology

Once collected triceps muscle samples were immediately frozen by immersion in pre-chilled 2-methyl butane (isopentane), then embedded in a cryoprotective medium and stored at −80 °C until sectioning. Transverse cryosections (10 µm thick) were obtained at −20 °C using a cryostat (Leica Biosystems CM3050 S, Nussloch, Germany). Muscle morphology was evaluated by hematoxylin and eosin (H&E) staining, following established histological protocols. To ensure consistency across samples, only transverse muscle sections were included in the analysis. Longitudinal sections, identified by alternating light and dark sarcomeric bands, were excluded. Transverse sections were confirmed by the presence of fascicular organization and identifiable endomysium and perimysium structures. Micrographs of randomly selected non-overlapping fields per sample and per animal were captured using a Leica microscope equipped with the LAS V4.0 software. To minimize any bias, all analyses were performed blinded to treatment.

### 2.13. Statistical Analysis

Data are presented as mean ± standard error of the mean (SEM). Statistical analyses were performed using the GraphPad Prism 7 software (GraphPad software, San Diego, CA, USA). For comparisons involving more than two independent groups, data were analyzed using the Kruskal–Wallis test, followed by the Dunn’s tests for multiple comparisons. Wilcoxon signed-rank test was used to compare two paired samples. A *p*-value < 0.05 was considered statistically significant.

## 3. Results

Previous studies from our group, and other research groups, established that CIS administration induces a wide range of deleterious effects in animal models, including oxidative stress, cognitive impairment, and muscle weakness [42,43]. NAC, a well-known antioxidant, has been shown to attenuate several of these adverse outcomes, likely through diverse mechanisms involving the restoration of redox balance and the reduction in ROS levels [44,45]. In our previous work, we demonstrated that coadministration of NAC with CIS not only mitigated oxidative and cognitive impairments but also resulted in a significant improvement in muscle performance. However, it remained unclear whether this improvement in muscle function reflected a direct protective effect on skeletal muscle tissue or whether it was secondary to improved cognitive or motivational states.

To resolve this ambiguity, we implemented complementary muscle-specific functional assays, designed to accurately evaluate muscle strength. Additionally, we explored potential molecular and redox-related mechanisms that might underlie the myoprotective effects of NAC in the context of CIS-induced toxicity.

It is worth noting that most previous studies investigating CIS-induced myotoxicity have used male rodents, whereas data on females are scared. Given that CIS is widely used in the treatment of solid tumors, including breast cancer, which predominantly affects females, we selected female Wistar rats to explore whether the myotoxic effects of CI show sex-specific difference and to expand the current knowledge on this subject.

### 3.1. NAC Administration Prevents CIS-Induced Decline in Muscle Strength

**Figure 1:**
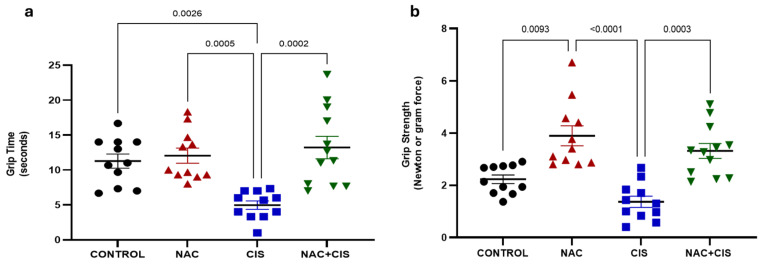
Effect of N-acetylcysteine (NAC) co-administration with cisplatin (CIS) on hangtime (**a**) and forelimb grip strength (**b**). Data are presented as mean ± SEM (*n* = 11–12 per group). *p*-values based on Kruskal–Wallis test, followed by Dunn’s tests.

Upon completion of the treatment protocol, muscle strength was assessed using two complementary approaches. The first method was the wire hang test (Figure 1a), which indirectly measures forelimb strength based on the time that the animal can maintain its grip on a horizontal bar. While this assay reflects muscular performance, it can also be influenced by non-muscular factors such as motivation, stress resilience, or depressive-like behaviors. Therefore, a complementary strategy was employed using a digital dynamometer (Figure 1b), which provides a quantitative and direct measurement of the pulling force exerted by an animal’s forelimbs.

In the wire hang test, rats treated with CIS displayed a significant reduction (~50%) in hang latency compared to control animals, suggesting compromised muscle performance. Remarkably, this effect was reversed in the NAC + CIS group, where hang latency values were restored to levels comparable to those of the control group, indicating a protective role for NAC.

In the dynamometry assessment, CIS treatment resulted in a marked decline in forelimb grip force (1.37 ± 0.22 N vs. Control: 2.23 ± 0.16 N), confirming muscle weakness in this group. However, co-administration of NAC significantly improved grip strength (NAC + CIS: 3.31 ± 0.28 N), demonstrating a robust myoprotective effect of NAC against CIS-induced muscular atrophy.

Interestingly, NAC administration resulted in increased grip strength compared to control animals, despite the absence of muscle mass gain. This finding is in line with reports in muscular dystrophy models, where NAC enhanced muscle performance via improvements in redox balance, neuromuscular adaptations and decreased inflammation [46,47].

### 3.2. NAC Prevents CIS-Induced Skeletal Muscle Atrophy and Structural Damage

**Figure 2:**
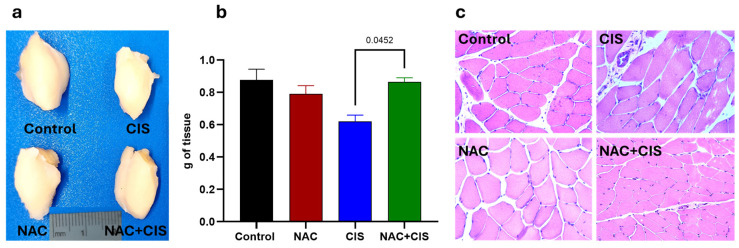
Effect of NAC co-administration with CIS on skeletal muscle mass and histological structure. Representative macroscopic images of dissected triceps from each group (**a**); quantification of muscle wet weight (in grams) of the experimental groups (**b**); and representative micrographs of muscle cross-sections stained with hematoxylin and eosin (H&E) captured at magnification of 40× (**c**). Data are presented as mean ± SEM (*n* = 5). *p*-values based on the Kruskal–Wallis test followed by Dunn’s tests.

Following functional muscular assessments, skeletal muscle tissue (triceps) was dissected to evaluate the impact of the treatments on muscle mass and histology integrity. As shown in Figure 2a,b, CIS-treated animals showed a significant reduction in triceps muscle weight compared to control ones, which is consistent with chemotherapy-induced muscle atrophy, a hallmark of cancer cachexia and systemic toxicity. Interestingly, co-administration of NAC prevented the loss of muscle mass, suggesting a protective effect against CIS.

Histological examination of muscle cross-sections provided further insights into the structural effects of the used treatments. In this sense, muscles from CIS-treated rats showed marked structural alterations, such as elongation and deformation, resulting in the loss of the characteristic hexagonal morphology of muscle fibers and therefore in the disorganization of their normal angular pattern, compared to control group’s tissues. Additionally, a visible reduction in muscle fiber density was observed in CIS-treated animals, potentially indicating fiber degeneration or impaired regeneration as a result of muscle damage caused by a chemotherapeutic insult. In contrast, muscle samples from the NAC + CIS group showed partial restoration of tissue architecture with a noticeably higher density of muscle fibers compared to the CIS group (Figure 2c). However, despite this improvement, residual abnormalities in fiber orientation and geometry persisted, such as the loss of typical angulation, suggesting that while NAC effectively attenuates structural damage, complete restoration of muscle integrity may require extended recovery time.

### 3.3. NAC Co-Administration Modulates Redox Imbalance Induced by CIS in Skeletal Muscle

After establishing that NAC prevents skeletal muscular dysfunction induced by CIS, we decided to explore the underlying mechanism of myoprotection, focusing on the role of oxidative stress, a key contributor to muscle atrophy during chemotherapy [48,49]. As oxidative stress alters cellular redox homeostasis, our initial objective was to determine the intracellular redox status by determining the GSH/GSSG ratio. In addition, we evaluated the activity of two key antioxidant enzymes involved in glutathione metabolism: glutathione peroxidase (GPx) and glutathione reductase (GR).

**Figure 3:**
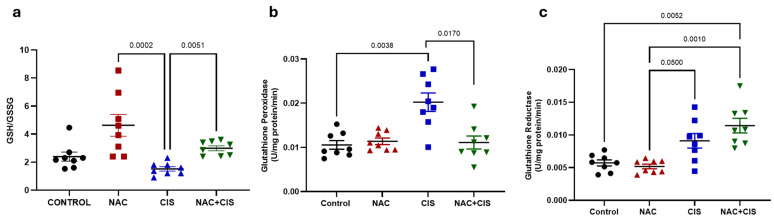
Effect of NAC-co-administration on CIS-induced GSH/GSSG imbalance in skeletal muscle. GSH/GSSG ratio in triceps homogenates across experimental groups (**a**); Glutathione peroxidase (GPx) activity (**b**) and glutathione reductase activity (**c**). Data are presented as mean ± SEM (*n* = 8 per group). *p*-values based on the Kruskal–Wallis test, followed by Dunn’s tests.

As shown in Figure 3a, CIS treatment tended to present a reduced GSH/GSSG ratio (~40%) compared to the control group, indicating an oxidative imbalance, consistent with previous reports regarding CIS toxicity [50]. In contrast, animals treated only with NAC alone displayed a trend toward an increased GSH/GSSG ratio (~1-fold increase vs. CIS), suggesting an enhanced antioxidant capacity. Most notably, in the NAC + CIS group, the GSH/GSSG ratio was significantly higher than the CIS group (~1-fold increase), indicating a possible partial restoration of redox homeostasis by NAC.

Interestingly, CIS administration resulted in a significant upregulation of antioxidant enzymes activity, with an increase in GPx and GR activities around 90 and 60%, respectively, compared to control group (Figure 3b,c). When NAC was co-administered with CIS, a significant reduction in GPx activity was observed compared to the CIS group, approaching control values. In contrast, GR activity remained elevated, similar to the levels observed in CIS-treated animals.

**Figure 4:**
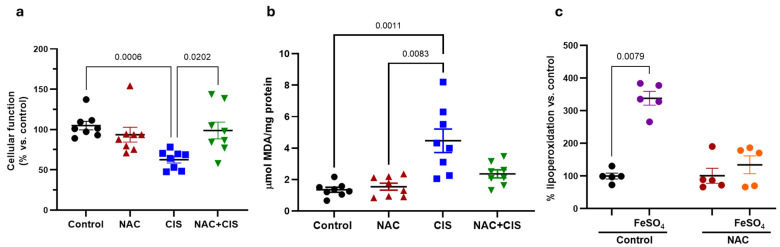
Effect of NAC on cellular function and lipid peroxidation alterations induced by CIS in skeletal muscle. Cellular function assessed by MTT reduction (**a**); lipid peroxidation in triceps muscle homogenates (**b**); and ex vivo exposure of muscle homogenates from control and NAC-treated animals to FeSO_4_ (**c**). Data are presented as mean ± SEM (*n* = 8 per group for a and b; *n* = 5 for ex vivo experiment). In (**a**,**b**) *p*-values are based on the Kruskal–Wallis test, followed by Dunn’s tests. In (**c**) *p*-value is based on the Wilcoxon signed rank test.

Given that NAC co-administration partially restores redox balance in skeletal muscle, we next sought to determine whether this redox improvement translated into enhanced cellular functionality and reduced lipid peroxidation, the latter being as a secondary marker of oxidative damage. As expected, CIS administration showed a significant reduction in cellular function and, a notable increase in lipoperoxidation (Figure 4a and 4b, respectively). Importantly, co-administration of NAC with CIS attenuated both the decline in cellular function and the increase in lipid peroxidation, corroborating that the antioxidant effect of NAC helped to mitigate oxidative damage and to preserve tissue viability.

Considering that NAC alone modulated redox parameters and that its administration preceded CIS treatment by three days and, possibly pre-modulated redox parameters, we hypothesized that early NAC exposure could be preconditioning skeletal muscle tissue, enhancing its resistance to subsequent oxidative damage. To test this hypothesis, we performed an ex vivo oxidative challenge using muscle homogenates from control and NAC groups. Samples were incubated with ferrous sulfate (FeSO_4_, a well-established prooxidant agent), and lipid peroxidation levels were assessed thereafter. As shown in Figure 4c, FeSO_4_ exposure induced approximately a 2.5-fold increase in lipid peroxidation in control muscle homogenates, while those from NAC-treated animals did not exhibit a significant increase, suggesting that NAC pre-treatment induced a state of oxidative resilience in skeletal muscle.

### 3.4. Effect of NAC and CIS Administration on Mitochondrial DNA Content and Biogenesis-Related Proteins

**Figure 5:**
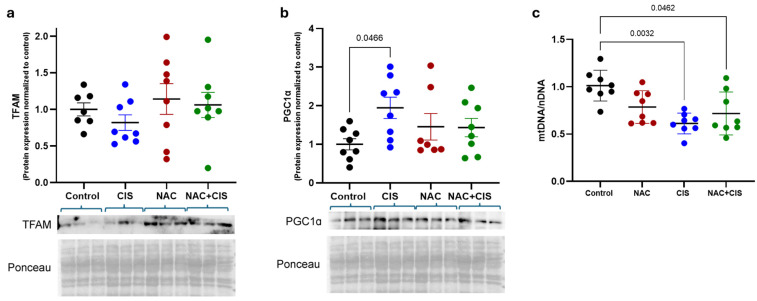
Effect of NAC and CIS administration on mitochondrial biogenesis markers and DNA content. TFAM (**a**) and PGC-1α (**b**) protein levels; mtDNA/nDNA ratio in triceps muscle (**c**). Data are presented as mean ± SEM (*n* = 7–8 per group). *p*-values based on the Kruskal–Wallis test followed by Dunn’s tests.

Considering that NAC may induce a protective or resilient cellular state, possibly through modulating mitochondrial biogenesis, we next evaluated two key transcriptional regulators of this process: peroxisome proliferator-activated receptor gamma coactivator-1 alpha (PGC-1α) and mitochondrial transcription factor A (TFAM) at the protein level. In parallel, we quantified the mtDNA/nDNA ratio, a commonly used indicator of mitochondrial content and functional biogenesis status [51]. As shown in Figure 5a,b, both TFAM and PGC-1α protein levels showed a non-significant upward trend in the NAC-treated group compared to control, suggesting a potential early activation of mitochondrial biogenesis signaling. These modest changes could reflect the initial stages of mitochondrial adaptation, which may require a longer exposure time to become functionally evident. Interestingly, CIS treatment significantly increased PCG-1α protein levels, a finding that could represent a compensatory cellular response to mitochondrial dysfunction and oxidative stress induced by this chemotherapeutic. However, no significant changes were detected in the NAC + CIS group for either TFAM or PCG-1α.

The analysis of the mtDNA/nDNA ratio revealed that CIS administration significantly reduced mitochondrial DNA content relative to nuclear DNA, consistent with mitochondrial loss or impaired replication due to chemotherapeutic-induced oxidative stress (Figure 5c). Interestingly, NAC co-administration failed to restore the mtDNA/nDNA ratio, suggesting that although NAC exerts antioxidant and structural effects, it may not fully rescue mitochondrial biogenesis, at least at the evaluated time.

### 3.5. Effect of NAC and CIS Administration on the Expression of Myogenic microRNA (myomiRs) and Their Target Genes

**Figure 6:**
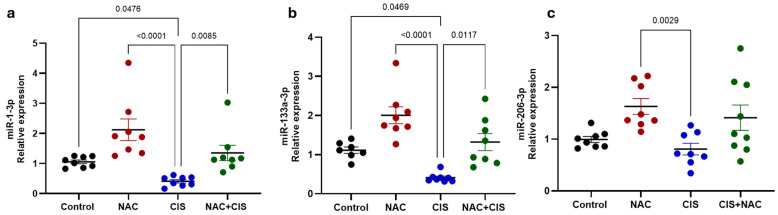
Relative expression of myomiRs miR-1-3p (**a**), miR-133a-3p (**b**) and miR-206-3p (**c**) across experimental groups. U6 was used as an internal control to normalize myomiRs’ expression. Data are presented as mean ± SEM (*n* = 7–8 per group). *p*-values based on the Kruskal–Wallis test followed by Dunn’s tests.

Since miRNAs have been identified as being involved in muscle development and atrophy, here we investigated the dysregulation of miRNAs in the context of skeletal muscle cisplatin-induced atrophy and NAC treatment. Since the protective effects of NAC appeared only partially depend on mitochondrial biogenesis, we next investigated whether the expression of three miRNAs with a key role in muscle differentiation, maintenance and regeneration known as myomiRs was affected. As shown in Figure 6a,b, administration of CIS significantly decreased the expression of miR-1-3p and miR-133a-3p compared to the control group. In contrast, NAC administration alone resulted in a trend toward increased expression of all three miRNAs, when compared to the controls; however, the three analyzed miRNAs were significantly upregulated in muscle of NAC administered rats in comparison to that of CIS-administered ones, which could result in muscle genesis or regeneration through the downregulation of some of their target genes. Remarkably, in the NAC + CIS group, the expression levels of miR-1-3p and miR-133-3p were restored to neat-control values, indicating that NAC co-administration preserves myogenic signaling pathways otherwise suppressed by CIS. Although miR-206-3p expression is not restored by NAC co-administration, a mild upward trend was observed when compared to the CIS group (Figure 6c).

**Figure 7:**
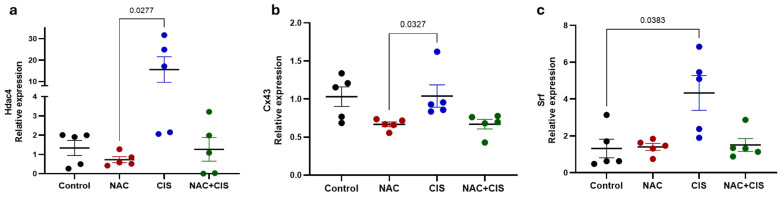
Relative expression of myomiRs’ target genes *Hdac4* (**a**), *Cx43* (**b**) and *Srf* (**c**) across experimental groups. β-actin was used as an internal control to normalize mRNAs’ expression. Data are presented as mean ± SEM (*n* = 5 per group). *p*-values based on the Kruskal–Wallis test followed by Dunn’s tests.

As previously mentioned, miRNAs usually downregulate their target genes either by messenger RNA (mRNA) degradation or by translational inhibition [52]. Since muscle differentiation requires an exquisitely regulated balance between the number of proliferating muscle stem cells (myoblasts) and differentiating myotubes, we first decided to measure the expression of the muscle genes’ repressor histone deacetylase 4 (*Hdac4*) which is known to be directly targeted by miR-1 to induce myogenesis [53]. As observed in Figure 7a, *Hdac4* presented an inverse expression profile to that observed for its regulator myomiR, miR-1-3p (Figure 6a); as it is significantly downregulated in response to NAC and also presented a trend to be less expressed in the NAC + CIS group when compared to CIS-treated rats, suggesting that miR-1-3p possibly promotes myogenesis at least in part by negatively regulating *Hdac4* expression in NAC presence. Additionally, we analyzed the expression of the gap-junction protein connexin 43 (*Cx43*) which mediates skeletal myoblast fusion during the initial stages of muscle development and regeneration; but thereafter it must be inhibited by miR-1 and miR-206 for complete myoblast differentiation to occur [54]. Similarly to *Hdac4*, *Cx43* was significantly upregulated in the muscle of CIS-treated animals when compared to the levels observed in the NAC-treated group (Figure 7b). This is consistent with NAC-induced myogenesis and the observed downregulation of its two negative regulators miR-1-3p (Figure 6a) and miR-206-3p (Figure 6c) between that NAC- and the CIS-administered groups. Finally, we analyzed the expression of the serum response factor (*Srf*) which is targeted by miR-133 to enhance myoblasts’ proliferation during muscle development [53]. As observed in Figure 7c, despite a trend for *Srf* to be less expressed in NAC and NAC + CIS groups when compared to CIS-administered rats, the only statistically significant change in its expression was observed between the control and the CIS-treated groups (Figure 7c). This suggest that in contrast to miR-1-3p and miR-206-3p, in our model miR-133a-3p may guarantees the survival and availability of muscle stem cells whose presence is key for muscle regeneration in response to CIS. Moreover, it is important to emphasize that miRNAs can also downregulate the protein level of their target genes, thus, it is possible that some of the observed trends in mRNA expression become statistically significant at the protein level.

## 4. Discussion

Cancer remains one of the leading health challenges worldwide, and despite advances in targeted therapies and immunotherapeutics, platinum-based chemotherapy such as CIS continues to be a standard the treatment of solid tumors. However, its clinical efficacy is often compromised by dose-limiting toxicities, including nephrotoxicity, ototoxicity, neurotoxicity and chemotherapy-induced cachexia. As we mentioned before, cachexia is a multifactorial syndrome characterized by skeletal muscle atrophy and functional decline that impacts patient quality of life and treatment tolerance [13,17].

In a separate study about tryptophan metabolites and CIS-induced cognitive dysfunction, our group previously reported that NAC, initially used as an enzyme inhibitor, unexpectedly improved grip strength in CIS-treated animals. This observation prompted the present study, where we systematically evaluated the myoprotective and myogenic potential of NAC against CIS-induced muscle atrophy and explored its potential redox and molecular mechanisms.

Consistent with prior studies [13,55], we observed that CIS administration significantly decreased muscle strength and skeletal muscle mass, accompanied by histological evidence of fiber disorganization and cellular degeneration. Notably, here we showed that NAC co-administration reversed these deficits and improved functional outcomes beyond control levels, suggesting both a protective and a possible ergogenic effect under physiological conditions. Despite the absence of significant changes in muscle mass. NAC-treated animals showed improved grip strength. This decoupling between muscle mass and function has been previously observed and attributed to improved oxidative status, mitochondrial function and neuromuscular performance [46].

While most prior research with NAC has focused on nephro- and neuroprotection in the context of CIS toxicity [20,56,57], our findings represent, to our knowledge, the first detailed report of NAC directly protecting skeletal muscle from CIS-induced myotoxicity. CIS-induced cachexia results from a complex set of cellular mechanisms that includes the induction of oxidative stress. Given the widely described role of NAC as a modulator of redox homeostasis, and as a precursor of GSH, we first explored whether its myoprotective effects were mediated through antioxidant pathways. Our data showed that CIS induces a decline in the GSH/GSSG ratio, consistent with oxidative stress presence, while NAC improved this parameter, correlating with an enhanced cellular function while reducing lipid peroxidation. GPx and GR are key enzymes in the cellular antioxidant defense system driven by GSH and in ROS scavenging. It has been previously shown that NAC pretreatment can restore the GPx and GR activities reduced by oxidative stress. Our results showed that CIS induced an increase in GPx and GR activities, likely as a compensatory response to restore redox balance, whereas NAC normalized GPx activity and maintained GR elevation, a pattern that may reflect a coordinated rebalancing of redox enzymatic systems. Particularly considering that pre-treatment with NAC could first elevate the intracellular levels of GSH and prepare cells for an increase in ROS induced by CIS treatment. Preconditioning against oxidative stress was tested in our ex vivo challenge with FeSO_4_ and NAC pretreatment, confirming that NAC not only mitigates oxidative stress in vivo, but also appears to induce a resilience phenotype, whereby tissues preconditioned with NAC exhibit greater resistance to secondary oxidative insults. This concept of NAC-induced cellular “preconditioning” has been observed in other systems, including the central nervous system, where it enhances resistance to cognitive disruptors [58].

As shown by the MTT assay, cytotoxicity exerted by CIS was also ameliorated by NAC in muscle tissues indicating that the mitochondrial activity of these cells is maintained by NAC since MTT conversion is mainly performed by mitochondrial enzymes like succinate dehydrogenases and oxidoreductases [59]. In addition to maintaining this NAC-induced metabolic integrity in muscle tissues, we next explored its effect in mitochondrial biogenesis, given its essential role in muscle maintenance and adaptation. Although no statistically significant differences were observed in TFAM and PGC-1α protein levels, the NAC-treated group showed a consistent upward trend, suggesting a potential early activation of mitochondrial biogenesis signaling. Unexpectedly, we observed increased PGC-1α protein levels in the CIS group. We hypothesize that this upregulation represents an early compensatory response to oxidative damage induced by CIS. This is supported by increased GPx and GR activity in the same group and is consistent with findings that acute ROS elevations can transiently increase PGC-1α expression [60,61]. However, the CIS-induced reduction in the mt-DNA/nDNA ratio was not reversed by NAC, suggesting that while NAC modulates upstream regulators, it may not fully rescue mitochondrial content under this study’s experimental conditions.

Importantly, our results also suggest a novel regulatory axis involving the myogenic miRNAs (myomiRs) miR-1-3p, miR-133a-3p and miR-206-3p. These myomiRs, are preferentially expressed in striated muscle tissue and play essential roles for muscle differentiation, homeostasis and regeneration [62,63]. Among them miR-206 is particularly notable for being exclusively expressed in skeletal muscle and is critically involved in muscle repair and satellite cell dynamics [64]. In this study, we demonstrated for the first time that CIS administration decreases the expression of these key myomiRs resulting in increased expression of some of their target genes which have a crucial role for myogenesis and muscle regeneration. Previous studies have shown that miR-1 and miR-206 are transiently downregulated in response to muscle injury, with their expression increasing during the transition from proliferating myoblasts to differentiated muscle cells or myocyte [53,65,66]. Their dynamic modulation is essential to allow the expansion of the satellite cell pool early in regeneration, followed by timely differentiation [65,67]. Mechanistically, both miR-1 and miR-206 share several target genes including the transcription factors paired box (Pax) 3 (*Pax3*) and *Pax7*, required for satellite cell proliferation, and the aforementioned *Hdac4* and *Cx43*. The repression of *Pax3*, *Pax7*, *Hdac4* and *Cx43* by these miRNAs facilitates the transition toward myogenic differentiation, underscoring their role in orchestrating muscle regeneration [64,65]. In the context of our study, the observed downregulation of myomiRs following CIS treatment is consistent with a disruption of this regenerative program. Importantly, co-administration of NAC restores the expression levels of miR-1-3p and miR-133a-3p, and partially recovered miR-206-3p, suggesting that NAC may not only act as an antioxidant but also as a modulator of myogenic regenerative signaling at the post-transcriptional level [68], which is consistent with the observed changes in the expression levels of the measured target genes. These findings support the hypothesis that NAC enhances muscle resilience during chemotherapy by preserving myomiRs-mediated regulatory gene networks, which are critical for maintaining muscle architecture and promoting recovery after injury.

Interestingly, the myoprotective effect of NAC observed in our study aligns with findings from other preclinical models of muscle pathology. In a study using the dy2J/dy2J mouse model of muscular dystrophy, characterized by chronic oxidative stress and mitochondrial dysfunction, NAC treatment(150 mg/kg IP/6 days/week for 22 days) prevented loss in muscle strength and showed a reduction in muscle fiber degeneration and central nucleation as well as decreased ROS levels in muscle tissue, suggesting that NAC contributes to the preservation of muscular integrity by mitigating oxidative stress and maintaining redox homeostasis [69]. Supporting this idea, another study using an acute compartment syndrome model, a condition characterized by ischemia–reperfusion injury and oxidative damage, reported that NAC administration reduce ROS, enhance muscle function, and reduce tissue fibrosis [44].

Taken together, our findings provide compelling evidence that NAC exerts multimodal protective effects on skeletal muscle during CIS chemotherapy. These are mediated, at least in part, through the restoration of redox balance, the modulation of antioxidant enzymatic activity, an early activation of mitochondrial biogenesis pathways and the preservation of the expression of essential miRNAs for muscle integrity. Nevertheless, it is important to consider that other mechanisms could also be involved in the positive effects of NAC, such as a possible antiapoptotic effect, as it has been previously reported in models of sarcopenia due to chronic liver disease [70].

Cachexia affects 40–80% of all advanced cancer patients and is associated with reduced response to chemotherapy, increased treatment toxicity, and poor prognosis. To date, no treatment has been approved for clinical use in this condition. A few investigations have been proposed to mitigate the debilitating effects of cachexia, including exercise and pharmaceutical adjuvant interventions. Adjuvant drug candidates include activin receptor signaling inhibitors, appetite stimulants, nutritional supplements and phytotherapies [57]. Since mitochondrial dysfunction and oxidative stress are common features preceding cachectic myopathy, a well-known antioxidant and safe over-the-counter drug such as NAC represents a promising treatment for preventing this myopathy.

Even though our findings strongly support the myoprotective effects of NAC during CIS chemotherapy, some limitations were present. First, both the duration and dosage of NAC and CIS. The short-term treatment window may have limited the extent of mitochondrial biogenesis and structural regeneration take place. Second, the present study did not explore the long-term effects of NAC or CIS administration. This represents a key limitation, especially considering that NAC’s myogenic properties could potentially interfere with the antitumoral efficacy of CIS by enhancing cellular recovery or proliferation. Our group is currently performing ongoing studies in tumor-bearing animals to delineate NAC tissue selectivity and identify dosing regimens that maximize protection of non-malignant tissues while preserving chemotherapeutic potency. Future studies could also evaluate NAC treatment in combination with exercise or nutritional strategies, and also to investigate whether myomiRs can serve as circulating biomarkers of chemotherapy-induced myopathy, thus an early clinical intervention could be performed to prevent a poor prognosis.

## 5. Conclusions

Our findings demonstrated that NAC exerts a multimodal protective effect against skeletal muscle dysfunction induced by CIS. This protection is mediated through (1) restoration of redox homeostasis and reduction in cytotoxicity, (2) induction of oxidative resilience, and (3) preservation of key myomiRs and some of their associated target genes. These results position NAC as a promising adjuvant strategy to counteract the deleterious muscular side effects during chemotherapy.

## Figures and Tables

**Table 1:** Sequences of reverse transcription (RT) and qPCR primers.

Rat sncRNA/Gene	Transcript/miRNA ID	RT Primer	qPCR Primers
miR-1-3p	MIMAT0003125 in miRBase	GTCGTATCCA*GTGCAGGGTCCGAGG* *T*ATTCGCACTGGATACGAC**ATACAC**	Fwd: GGGCCTGGAATGTAAAGAAGT Rev: GTGCAGGGTCCGAGGT
miR-133a-3p	MIMAT0000839 in miRBase	GTCGTATCCA*GTGCAGGGTCCGAGG* *T*ATTCGCACTGGATACGAC**CAGCTG**	Fwd: GTGTTTGGTCCCCTTCAAC Rev: GTGCAGGGTCCGAGGT
miR-206-3p	MIMAT0000879 in miRBase	GTCGTATCCA*GTGCAGGGTCCGAGG* *T*ATTCGCACTGGATACGAC**CCACAC**	Fwd: GGCGTGGAATGTAAGGAAGT Rev: GTGCAGGGTCCGAGGT
U6	ENSG00000283249 in Ensembl	AACGCTTCACGAATTTGCGT	Fwd: CTCGCTTCGGCAGCACA Rev: AACGCTTCACGAATTTGCGT
*Cx43*	ENSRNOT00060013216.1 in Ensembl	N/A	Fwd: CTCGCCTATGTCTCCTCCTG Rev: TGTAGTTCGCCCAGTTTTGC
*Hdac4*	ENSRNOT00065034380.1 in Ensembl	N/A	Fwd: GTCTTGGGAATGTACGACG Rev: GCTCCGTCTCTCAGCTACTT
*Srf*	ENSRNOT00060044980.1 in Ensembl	N/A	Fwd: AGCCAGATCTCACCTACCAG Rev: GTGACTGTGAATGCTGGC
*β-actin*	ENSRNOT00000065528.2 in Ensembl	N/A	Fwd: CGTGCGGGATGTCAAAGAA Rev: AACGTTCGTTCCCAATGGTG

Note: A universal reverse primer was used for all miRNAs’ qPCR as it is complementary to a sequence contained within all stem-loop primers (labeled in italics and underlined). Bold letters in RT primers indicate a specific sequence for each single-stranded mature miRNA. For U6, the qPCR reverse primer was the same used for RT. For *Cx43*, *Hdac4*, *Srf*, and *β-actin* RT reactions, oligo (dT)15 and random hexamers were used. sncRNA: small non-coding RNA, N/A: not applicable.

## Data Availability

Data used to support the findings of this study are available with the corresponding author upon request.

## Abbreviations

The following abbreviations are used in this manuscript:

CIS	Cisplatin
Cx	Connexin
DNA	Deoxyribonucleic acid
FeSO_4_	Ferrous sulfate
GPx	Glutathione peroxidase
GR	Glutathione reductase
GSH	Glutathione reduced form
GSSG	Glutathione oxidized form
Hdac	Histone deacetylase
LP	Lipid peroxidation
MDA	Malondialdehyde
miRNAs	microRNAs
mtDNA	Mitochondrial DNA
myomiRs	Myogenic microRNAs
NAC	N-acetylcysteine
nDNA	Nuclear DNA
Pax	Paired box
PGC1α	Peroxisome Proliferator-Activated Receptor Gamma Coactivator 1-Alpha
ROS	Reactive oxygen species
Srf	Serum response factor
TFAM	Mitochondrial transcription factor A
